# Supplementary material for: All-cause and Infection-attributable Mortality Amongst Adults With Bloodstream Infection—a Population-based Study
Source: Open Forum Infect Dis. 2024 Mar 6;11(5):ofae126. doi: 10.1093/ofid/ofae126 (PMC11055210; doi:10.1093/ofid/ofae126)

**All-cause and infection-attributable mortality amongst adults with bloodstream infection – a population-based study**

Jonathan Underwood,^1,2^ Rowena Griffiths,^3^ David Gillespie,^4^ Ashley Akbari^3^ and Haroon Ahmed.^5^

1. Division of Infection and Immunity, Cardiff University, Cardiff, UK
2. Department of Infectious Diseases, Cardiff and Vale University Health Board, Cardiff, UK
3. Population Data Science, Swansea University, UK.
4. Centre for Trials Research, Cardiff University School of Medicine, Cardiff, UK
5. Division of Population Medicine, Cardiff University School of Medicine, Cardiff, UK

# Supplementary data

## Strobe checklist

|  | Item No. | Recommendation | Page  No. |
| --- | --- | --- | --- |
| **Title and abstract** | 1 | (*a*) Indicate the study’s design with a commonly used term in the title or the abstract | 2 |
|  |  | (*b*) Provide in the abstract an informative and balanced summary of what was done and what was found | 2 |
| Introduction | | | |
| Background/rationale | 2 | Explain the scientific background and rationale for the investigation being reported | 3 |
| Objectives | 3 | State specific objectives, including any prespecified hypotheses | 3 |
| Methods | | | |
| Study design | 4 | Present key elements of study design early in the paper | 3-4 |
| Setting | 5 | Describe the setting, locations, and relevant dates, including periods of recruitment, exposure, follow-up, and data collection | 3-4 |
| Participants | 6 | (*a*) *Cohort study*—Give the eligibility criteria, and the sources and methods of selection of participants. Describe methods of follow-up  *Case-control study*—Give the eligibility criteria, and the sources and methods of case ascertainment and control selection. Give the rationale for the choice of cases and controls  *Cross-sectional study*—Give the eligibility criteria, and the sources and methods of selection of participants | 3-4 |
|  |  | (*b*) *Cohort study*—For matched studies, give matching criteria and number of exposed and unexposed  *Case-control study*—For matched studies, give matching criteria and the number of controls per case |  |
| Variables | 7 | Clearly define all outcomes, exposures, predictors, potential confounders, and effect modifiers. Give diagnostic criteria, if applicable | 4-5 |
| Data sources/ measurement | 8* | For each variable of interest, give sources of data and details of methods of assessment (measurement). Describe comparability of assessment methods if there is more than one group | *4-5* |
| Bias | 9 | Describe any efforts to address potential sources of bias | 5 |
| Study size | 10 | Explain how the study size was arrived at | 3-4 |

| Quantitative variables | 11 | Explain how quantitative variables were handled in the analyses. If applicable, describe which groupings were chosen and why | 4-5 |
| --- | --- | --- | --- |
| Statistical methods | 12 | (*a*) Describe all statistical methods, including those used to control for confounding | 5 |
|  |  | (*b*) Describe any methods used to examine subgroups and interactions | 5 |
|  |  | (*c*) Explain how missing data were addressed | 5 |
|  |  | (*d*) *Cohort study*—If applicable, explain how loss to follow-up was addressed  *Case-control study*—If applicable, explain how matching of cases and controls was addressed  *Cross-sectional study*—If applicable, describe analytical methods taking account of sampling strategy | 3-4 |
|  |  | (*e*) Describe any sensitivity analyses | 5 |
| Participants | 13* | (a) Report numbers of individuals at each stage of study—eg numbers potentially eligible, examined for eligibility, confirmed eligible, included in the study, completing follow-up, and analysed | 5-6 |
|  |  | (b) Give reasons for non-participation at each stage |  |
|  |  | (c) Consider use of a flow diagram |  |
| Descriptive data | 14* | (a) Give characteristics of study participants (eg demographic, clinical, social) and information on exposures and potential confounders | 5-6, table 1 |
|  |  | (b) Indicate number of participants with missing data for each variable of interest | Table 1 |
|  |  | (c) *Cohort study*—Summarise follow-up time (eg, average and total amount) | 6 |
| Outcome data | 15* | *Cohort study*—Report numbers of outcome events or summary measures over time | *6-7, tables 2-3* |
|  |  | *Case-control study—*Report numbers in each exposure category, or summary measures of exposure |  |
|  |  | *Cross-sectional study—*Report numbers of outcome events or summary measures |  |
| Main results | 16 | (*a*) Give unadjusted estimates and, if applicable, confounder-adjusted estimates and their precision (eg, 95% confidence interval). Make clear which confounders were adjusted for and why they were included | *6-7, tables 2-3* |
|  |  | (*b*) Report category boundaries when continuous variables were categorized |  |
|  |  | (*c*) If relevant, consider translating estimates of relative risk into absolute risk for a meaningful time period |  |

## Methods

The Office for National Statistics, which provides cause of death data in this study, publishes extensive methodological information: <https://www.ons.gov.uk/peoplepopulationandcommunity/birthsdeathsandmarriages/deaths/methodologies/userguidetomortalitystatisticsjuly2017#cause-of-death-coding>

“*The death certificate used in England and Wales is compatible with that recommended by WHO. It is set out in two parts. Part I gives the condition or sequence of conditions leading directly to death, while Part II gives details of any associated conditions that contributed to the death but are not part of the causal sequence.*

*The selection of the underlying cause of death is based on ICD rules and is made from the condition or conditions reported by the certifier, as recorded on the certificate. The underlying cause of death is defined by WHO as the disease or injury that initiated the train of events directly leading to death or the circumstances of the accident or violence that produced the fatal injury.”*

*“****Selection and modification rules***

*The selection of the underlying cause of death is generally made from the condition or conditions entered in the lowest completed line of Part I of the Medical Certificate of Cause of Death (MCCD). If the death certificate has not been completed correctly - for example, if there is more than one cause on a single line with no indication of sequence or the conditions entered are not an acceptable causal sequence - it becomes necessary to apply one or more of the selection rules in the ICD-10.*

*Even where the certificate has been completed properly, there are particular conditions, combinations or circumstances when modification rules have to be applied to select the correct underlying cause of death. On some death certificates, for example, when two or more causes are listed and then linked together, these may point to another cause (not mentioned directly on the certificate) as underlying (an inferred underlying cause). This happens in a minority of cases and these are most commonly related to diseases of the circulatory system and late effects of cerebrovascular disease. In other cases, the underlying cause of death can be selected from Part II of the MCCD.*

*In summary, the purpose behind the selection and modification rules is to derive the most useful information from the death certificate and to do it uniformly so that data will be comparable between places and times and each death certificate produces one, and only one, underlying cause of death.*

***Underlying cause of death versus contributory causes***

*Coding rules ensure that each recorded item on the certificate is coded independently of all others on the same certificate. All mentioned causes (the underlying cause of death and any other causes that were mentioned on the death certificate as contributing to the death) have been coded routinely since 1993.*

*Most of the regular ONS mortality tables analyse the underlying cause of death, often referred to as deaths "due to" a particular cause. In some publications we also refer to deaths "involving" a particular cause. This is a broader category that includes all deaths that had the cause mentioned on the death certificate, whether as the underlying cause or a contributory cause*.”

Table m1. ICD-10 codes used to identify sepsis as cause of death.

ICD 10 codes containing sepsis/septicaemia or SIRS

| ALT_CODE | DESCRIPTION |
| --- | --- |
| A021 | Salmonella sepsis |
| A207 | Septicaemic plague |
| A227 | Anthrax sepsis |
| A267 | Erysipelothrix sepsis |
| A327 | Listerial sepsis |
| A40 | Streptococcal sepsis |
| A400 | Sepsis due to streptococcus, group A |
| A401 | Sepsis due to streptococcus, group B |
| A402 | Sepsis due to streptococcus, group D |
| A403 | Sepsis due to Streptococcus pneumoniae |
| A408 | Other streptococcal sepsis |
| A409 | Streptococcal sepsis, unspecified |
| A41 | Other sepsis |
| A410 | Sepsis due to Staphylococcus aureus |
| A411 | Sepsis due to other specified staphylococcus |
| A412 | Sepsis due to unspecified staphylococcus |
| A413 | Sepsis due to Haemophilus influenzae |
| A414 | Sepsis due to anaerobes |
| A415 | Sepsis due to other Gram-negative organisms |
| A418 | Other specified sepsis |
| A419 | Sepsis, unspecified |
| A427 | Actinomycotic sepsis |
| B377 | Candidal sepsis |
| O85X | Puerperal sepsis |
| R65 | Systemic Inflammatory Response Syndrome [SIRS] |
| R650 | Systemic Inflammatory Response Syndrome of infectious origin without organ failure |
| R651 | Systemic Inflammatory Response Syndrome of infectious origin with organ failure |
| R652 | Systemic Inflammatory Response Syndrome of non-infectious origin without organ failure |
| R653 | Systemic Inflammatory Response Syndrome of non-infectious origin with organ failure |
| R659 | Systemic Inflammatory Response Syndrome, unspecified |

Table m2. Leading causes of death – ONS groupings of ICD-10 codes.

| Table 1: Leading causes of death in England and Wales (revised 2016) | |
| --- | --- |
| ICD-10 codes | Cause of death groups |
| A00–A09 | Intestinal infectious diseases |
| A15–A19, B90 | Tuberculosis |
| A20, A44, A75–A79, A82–A84, A85.2, A90–A98, B50–B57 | Vector–borne diseases and rabies |
| A33–A37, A49.2, A80, B01, B02, B05, B06, B15, B16, B17.0, B18.0, B18.1, B26, B91, G14 | Vaccine-preventable diseases^1^ |
| A39, A87, G00–G03 | Meningitis and meningococcal infection |
| A40–A41 | Septicaemia |
| B20–B24 | Human immunodeficiency virus [HIV] disease |
| C00–C97 | Malignant neoplasms |
| C15 | Malignant neoplasm of oesophagus |
| C16 | Malignant neoplasm of stomach |
| C18–C21 | Malignant neoplasm of colon, sigmoid, rectum and anus |
| C22 | Malignant neoplasm of liver and intrahepatic bile ducts |
| C23-C24 | Malignant neoplasm of gallbladder and other parts of biliary tract |
| C25 | Malignant neoplasm of pancreas |
| C32 | Malignant neoplasm of larynx |
| C33-C34 | Malignant neoplasm of trachea, bronchus and lung |
| C40–C41 | Malignant neoplasms of bone and articular cartilage |
| C43-C44 | Melanoma and other malignant neoplasms of skin |
| C50 | Malignant neoplasm of breast |
| C53–C55 | Malignant neoplasm of uterus |
| C56 | Malignant neoplasm of ovary |
| C61 | Malignant neoplasm of prostate |
| C64 | Malignant neoplasm of kidney, except renal pelvis |
| C67 | Malignant neoplasm of bladder |
| C71 | Malignant neoplasm of brain |
| C81–C96 | Malignant neoplasms, stated or presumed to be primary of lymphoid, haematopoietic and related tissue |
| D00–D48 | In situ and benign neoplasms, and neoplasms of uncertain or unknown behaviour |
| E10–E14 | Diabetes |
| D50–D53, E40–E64 | Malnutrition, nutritional anaemias and other nutritional deficiencies |
| E86–E87 | Disorders of fluid, electrolyte and acid–base balance (incl. dehydration) |
| F01, F03, G30 | Dementia and Alzheimer disease |
| F10–F19 | Mental and behavioural disorders due to psychoactive substance use |
| G10–G12 | Systemic atrophies primarily affecting the central nervous system |
| G20 | Parkinson disease |
| G40-G41 | Epilepsy and status epilepticus |
| G80–G83 | Cerebral palsy and other paralytic syndromes |
| I05–I09 | Chronic rheumatic heart diseases |
| I10–I15 | Hypertensive diseases |
| I20–I25 | Ischaemic heart diseases |
| I26–I28 | Pulmonary heart disease and diseases of pulmonary circulation |
| I34–I38 | Nonrheumatic valve disorders and endocarditis |
| I42 | Cardiomyopathy |
| I46 | Cardiac arrest |
| I47–I49 | Cardiac arrhythmias |
| I50–I51 | Heart failure and complications and ill–defined heart disease |
| I60–I69 | Cerebrovascular diseases |
| I70 | Atherosclerosis |
| I71 | Aortic aneurysm and dissection |
| J00–J06, J20–J22 | Acute respiratory infections other than influenza and pneumonia |
| J09–J18 | Influenza and pneumonia |
| J40–J47 | Chronic lower respiratory diseases |
| J80–J84 | Pulmonary oedema and other interstitial pulmonary diseases |
| J96 | Respiratory failure |
| K35–K46, K56 | Appendicitis, hernia and intestinal obstruction |
| K70–K76 | Cirrhosis and other diseases of liver |
| M00–M99 | Diseases of the musculoskeletal system and connective tissue |
| N00–N39 | Diseases of the urinary system |
| O00–O99 | Pregnancy, childbirth and the puerperium |
| P00–P96 | Certain conditions originating in the perinatal period |
| Q00–Q99 | Congenital malformations, deformations and chromosomal abnormalities |
| V01–X59 | Accidents |
| V01–V89 | Land transport accidents |
| W00–W19 | Accidental falls |
| W32–W34 | Non-intentional firearm discharge |
| W65–W74 | Accidental drowning and submersion |
| W75–W84 | Accidental threats to breathing |
| X40–X49 | Accidental poisoning |
| X60–X84, Y10–Y34 | Suicide and injury/poisoning of undetermined intent^2^ |
| U50.9, X85–Y09, Y87.1 | Homicide and probable homicide |
| R00-R99 | Symptoms, signs and ill-defined conditions |
| Source: Office for National Statistics | |
| Notes: | |
| 1. Excluding meningitis and meningococcal diseases (A39), sepsis due to haemophilus influenzae (A41.3), rabies (A82), certain mosquito-borne diseases (A83) and yellow fever (A95). | |
| 2. In England and Wales, a conclusion of suicide cannot be returned for children under the age of 10 years. | |

## Results

Table s1. BSI demographics by number of BSI episodes

|  | **Number of BSI** | |  |
| --- | --- | --- | --- |
| **Variable** | **Single infection**, N = 31,259^1^ | **Multiple infections**, N = 4,432^1^ | **p-value**^2^ |
| **Age (years)** | 75 (63 – 83) | 75 (64 – 83) | 0.32 |
| **Sex** |  |  | <0.001 |
| *Female* | 14,911 (48) | 1,855 (42) |  |
| *Male* | 16,348 (52) | 2,577 (58) |  |
| **Frailty Rating** |  |  | <0.001 |
| *Fit* | 11,322 (36) | 1,395 (31) |  |
| *Mild* | 9,867 (32) | 1,448 (33) |  |
| *Moderate* | 7,009 (22) | 1,125 (25) |  |
| *Severe* | 3,061 (9.8) | 464 (10) |  |
| **Charlson Index** | 8 (0 – 19) | 9 (2 – 19) | <0.001 |
| **Organsim** |  |  | <0.001 |
| *E. coli* | 19,259 (62) | 2,011 (45) |  |
| *Klebsiella* | 3,312 (11) | 176 (4.0) |  |
| *MRSA* | 904 (2.9) | 53 (1.2) |  |
| *MSSA* | 6,070 (19) | 488 (11) |  |
| *Polymicrobial* | 597 (1.9) | 1,644 (37) |  |
| *PsA* | 1,117 (3.6) | 60 (1.4) |  |
| **Welsh Index of Multiple Deprivation** |  |  | 0.45 |
| *1* | 6,521 (22) | 979 (23) |  |
| *2* | 6,411 (22) | 927 (22) |  |
| *3* | 6,337 (21) | 877 (21) |  |
| *4* | 5,269 (18) | 745 (18) |  |
| *5* | 5,101 (17) | 706 (17) |  |
| *Missing* | 1,620 | 198 |  |
| **Peak CRP (mg/L)** | 205 (122 – 296) | 197 (119 – 281) | <0.001 |
| *Missing* | 3,119 | 354 |  |
| ^1^Median (IQR); n (%) | | | |
| ^2^Wilcoxon rank sum test; Pearson's Chi-squared test | | | |

Table s2. Characteristics of patients by timing and cause of death.

|  | **Timing of death** | | | **Cause of death** | | |
| --- | --- | --- | --- | --- | --- | --- |
| **Variable** | **Died 0-2 days**, N = 2,523^1^ | **Died 3-30 days**, N = 5,314^1^ | **p-value**^2^ | **Other causes**, N = 5,783^1^ | **Sepsis**, N = 2,054^1^ | **p-value**^2^ |
| **Age (years)** | 78 (68 – 85) | 79 (69 – 86) | <0.001 | 78 (69 – 86) | 79 (70 – 86) | 0.087 |
| **Sex** |  |  | 0.030 |  |  | 0.001 |
| *Female* | 1,126 (45) | 2,234 (42) |  | 2,418 (42) | 942 (46) |  |
| *Male* | 1,397 (55) | 3,080 (58) |  | 3,365 (58) | 1,112 (54) |  |
| **Frailty Rating** |  |  | 0.76 |  |  | 0.15 |
| *Fit* | 784 (31) | 1,641 (31) |  | 1,797 (31) | 628 (31) |  |
| *Mild* | 762 (30) | 1,655 (31) |  | 1,816 (31) | 601 (29) |  |
| *Moderate* | 681 (27) | 1,384 (26) |  | 1,500 (26) | 565 (28) |  |
| *Severe* | 296 (12) | 634 (12) |  | 670 (12) | 260 (13) |  |
| **Charlson Index** | 14 (4 – 24) | 17 (8 – 26) | <0.001 | 16 (8 – 26) | 14 (4 – 23) | <0.001 |
| **Organism** |  |  | <0.001 |  |  | <0.001 |
| *E. coli* | 1,327 (53) | 2,552 (48) |  | 2,888 (50) | 991 (48) |  |
| *Klebsiella* | 250 (9.9) | 568 (11) |  | 649 (11) | 169 (8.2) |  |
| *MRSA* | 105 (4.2) | 266 (5.0) |  | 255 (4.4) | 116 (5.6) |  |
| *MSSA* | 418 (17) | 1,268 (24) |  | 1,234 (21) | 452 (22) |  |
| *Polymicrobial* | 241 (9.6) | 440 (8.3) |  | 479 (8.3) | 202 (9.8) |  |
| *PsA* | 182 (7.2) | 220 (4.1) |  | 278 (4.8) | 124 (6.0) |  |
| **Welsh Index of Multiple Deprivation** |  |  | 0.24 |  |  | 0.93 |
| *1* | 509 (22) | 1,048 (21) |  | 1,139 (21) | 418 (21) |  |
| *2* | 507 (22) | 1,121 (22) |  | 1,186 (22) | 442 (23) |  |
| *3* | 508 (22) | 1,056 (21) |  | 1,150 (21) | 414 (21) |  |
| *4* | 410 (18) | 902 (18) |  | 964 (18) | 348 (18) |  |
| *5* | 373 (16) | 913 (18) |  | 956 (18) | 330 (17) |  |
| *Missing* | 216 | 274 |  | 388 | 102 |  |
| **Peak CRP (mg/L)** | 200 (101 – 308) | 236 (149 – 319) | <0.001 | 219 (127 – 310) | 249 (151 – 332) | <0.001 |
| *Missing* | 329 | 501 |  | 625 | 205 |  |
| ^1^Median (IQR); n (%) | | | | | | |
| ^2^Wilcoxon rank sum test; Pearson's Chi-squared test | | | | | | |

Abbreviations: MRSA: Methicillin-resistant *Staphylococcus aureus*; MSSA: Methicillin-sensitive *Staphylococcus aureus*; PsA: *Pseudomonas aeruginosa.*

Table s3. BSI mortality by organism for patients with only one recorded BSI.

|  | **Status at 30-days** | |  |
| --- | --- | --- | --- |
|  | Alive | Dead | **Total** |
| **Organism** |  |  |  |
| *E. coli* | 15,810 (82%) | 3,449 (18%) | 19,259 (100%) |
| *Klebsiella* | 2,540 (77%) | 772 (23%) | 3,312 (100%) |
| *MRSA* | 549 (61%) | 355 (39%) | 904 (100%) |
| *MSSA* | 4,484 (74%) | 1,586 (26%) | 6,070 (100%) |
| *Polymicrobial* | 403 (68%) | 194 (32%) | 597 (100%) |
| *PsA* | 739 (66%) | 378 (34%) | 1,117 (100%) |
| **Total** | 24,525 (78%) | 6,734 (22%) | 31,259 (100%) |

Table s4. E. coli BSI deaths with UTI as underlying cause of infection stratified by whether sepsis listed as a cause of death anywhere on the death certificate

| **Characteristic** | **Sepsis not mentioned**, N = 163^1^ | **Sepsis**, N = 155^1^ | **p-value**^2^ |
| --- | --- | --- | --- |
| **Age** | 84 (78 – 90) | 84 (78 – 90) | >0.99 |
| **Days survived** | 4.0 (2.0 – 11.0) | 5.0 (1.5 – 10.0) | 0.96 |
| **Sex** |  |  | 0.38 |
| Female | 75 (46) | 79 (51) |  |
| Male | 88 (54) | 76 (49) |  |
| **Frailty category** |  |  | 0.86 |
| Fit | 31 (19) | 26 (17) |  |
| Mild | 40 (25) | 44 (28) |  |
| Moderate | 57 (35) | 54 (35) |  |
| Severe | 35 (21) | 31 (20) |  |
| **CHARLSON_INDEX** | 14 (3 – 22) | 14 (4 – 24) | 0.65 |
| **WIMD** |  |  | 0.21 |
| 1 | 31 (21) | 47 (32) |  |
| 2 | 29 (19) | 23 (16) |  |
| 3 | 38 (26) | 35 (24) |  |
| 4 | 24 (16) | 24 (16) |  |
| 5 | 27 (18) | 18 (12) |  |
| Unknown | 14 | 8 |  |
| **Peak CRP (mg/L)** | 252 (160 – 336) | 262 (145 – 320) | 0.41 |
| Unknown | 12 | 20 |  |
| ^1^Median (IQR); n (%) | | | |
| ^2^Wilcoxon rank sum test; Pearson's Chi-squared test | | | |

Table s5. MSSA BSI deaths with endocarditis as the underlying cause of infection stratified by whether sepsis listed as a cause of death anywhere on the death certificate

| **Characteristic** | **Sepsis not mentioned**, N = 64^1^ | **Sepsis**, N = 20^1^ | **p-value**^2^ |
| --- | --- | --- | --- |
| **Age (years)** | 77 (70 – 83) | 74 (67 – 84) | 0.75 |
| **Days survived** | 13 (9 – 22) | 11 (6 – 22) | 0.53 |
| **Sex** |  |  | 0.71 |
| Female | 35 (55) | 10 (50) |  |
| Male | 29 (45) | 10 (50) |  |
| **Frailty category** |  |  | 0.89 |
| **CHARLSON_INDEX** | 13 (2 – 23) | 18 (8 – 22) | 0.57 |
| **WIMD** |  |  | 0.84 |
| **Peak CRP (mg/L)** | 264 (189 – 334) | 288 (166 – 332) | 0.87 |
| Unknown | <5 | <5 |  |
| ^1^Median (IQR); n (%) | | | |
| ^2^Wilcoxon rank sum test; Pearson's Chi-squared test; Fisher's exact test | | | |

Table s5. E. coli and MSSA first listed causes of death by ONS grouping

| **E. coli** | | | |
| --- | --- | --- | --- |
| <30 days | | 30-90 days | |
| *Miscellaneous** | 852 (22%) | Malignant neoplasms | 502 (13%) |
| **Sepsis** | **738 (19%)** | Influenza and pneumonia | 237 (6%) |
| Influenza and pneumonia | 603 (16%) | *Miscellaneous* | 232 (6%) |
| Malignant neoplasms | 593 (15%) | Diseases of the urinary tract^††^ | 95 (2%) |
| Diseases of the urinary tract^†^ | 371 (10%) | **Sepsis** | **87 (2%)** |
| Cirrhosis | 89 (2%) | Dementia | 69 (2%) |
| Ischaemic heart disease | 89 (2%) | Heart failure | 62 (2%) |
| Heart failure | 73 (2%) | Ischaemic heart disease | 51 (1%) |
| Cerebrovascular diseases | 64 (2%) | Frailty | 44 (1%) |
| COVID | 47 (1%) | Cerebrovascular diseases | 37 (1%) |
|  |  |  |  |
| **MSSA** | | | |
| <30 days | | 30-90 days | |
| *Miscellaneous**** | 371 (22%) | Malignant neoplasms | 97 (19%) |
| Influenza and pneumonia | 318 (19%) | *Miscellaneous***** | 96 (19%) |
| **Sepsis** | **308 (18%)** | Influenza and pneumonia | 67 (13%) |
| Malignant neoplasms | 150 (9%) | Heart failure | 39 (8%) |
| Heart failure | 96 (6%) | **Sepsis** | **37 (7%)** |
| Diseases of the urinary tract^†††^ | 79 (5%) | Diseases of the urinary tract^††††^ | 29 (6%) |
| Cerebrovascular diseases | 75 (4%) | Cerebrovascular diseases | 22 (4%) |
| Cirrhosis | 48 (3%) | Ischaemic heart disease | 19 (4%) |
| Ischaemic heart disease | 45 (3%) | Cirrhosis | 16 (3%) |
| COVID | 38 (2%) | Dementia | 12 (2%) |

* R68.8 “Other specified general symptoms and signs” n=282

** R68.8 “Other specified general symptoms and signs” n=75

*** R68.8 “Other specified general symptoms and signs” n=142

**** R68.8 “Other specified general symptoms and signs” n=24

^†^ N39 “Urinary tract infection” n=390

^††^ N39 “Urinary tract infection” n=58

^†††^ N39 “Urinary tract infection” n=33

^††††^ N39 “Urinary tract infection” n=9

Figure s1.


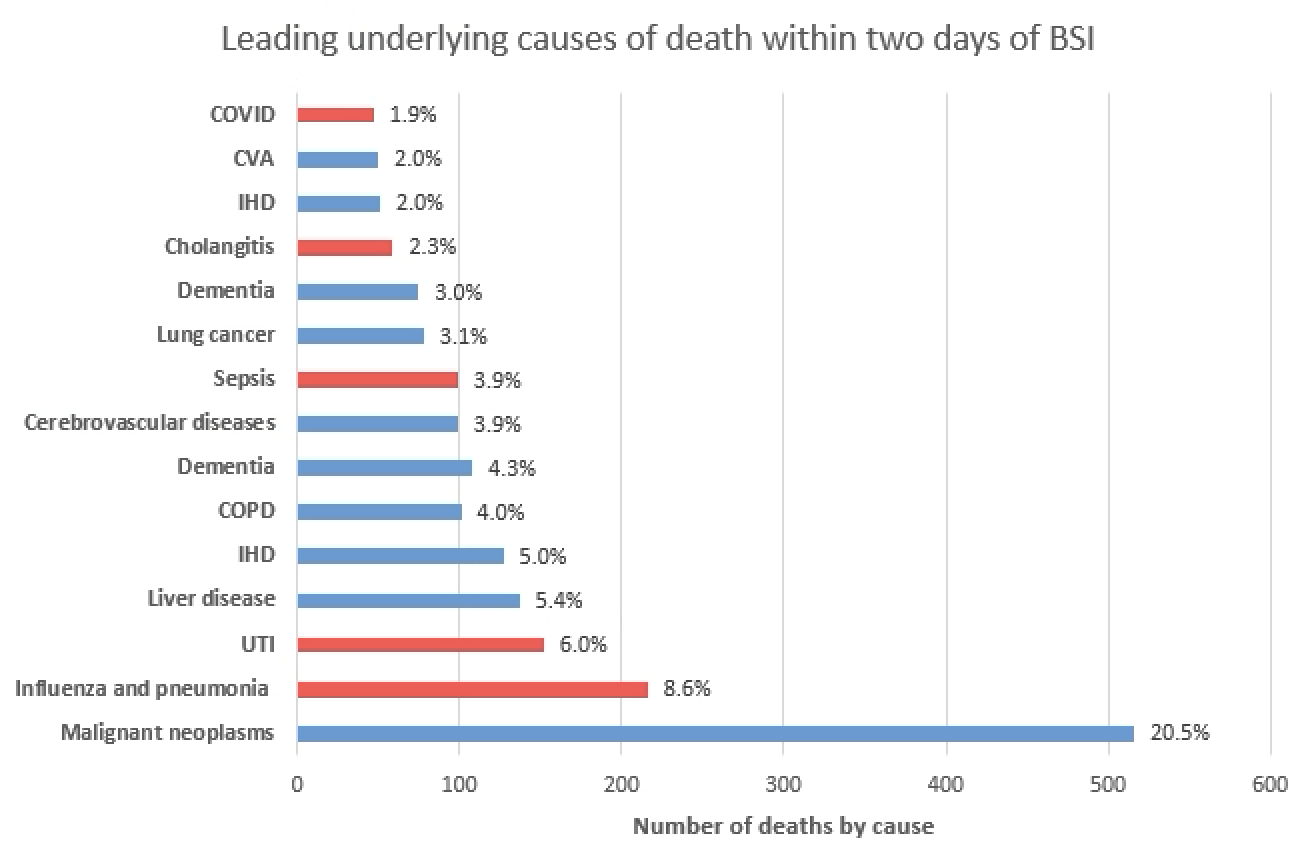


Figure s2. Timing of deaths involving sepsis within 30 days of bloodstream infection.


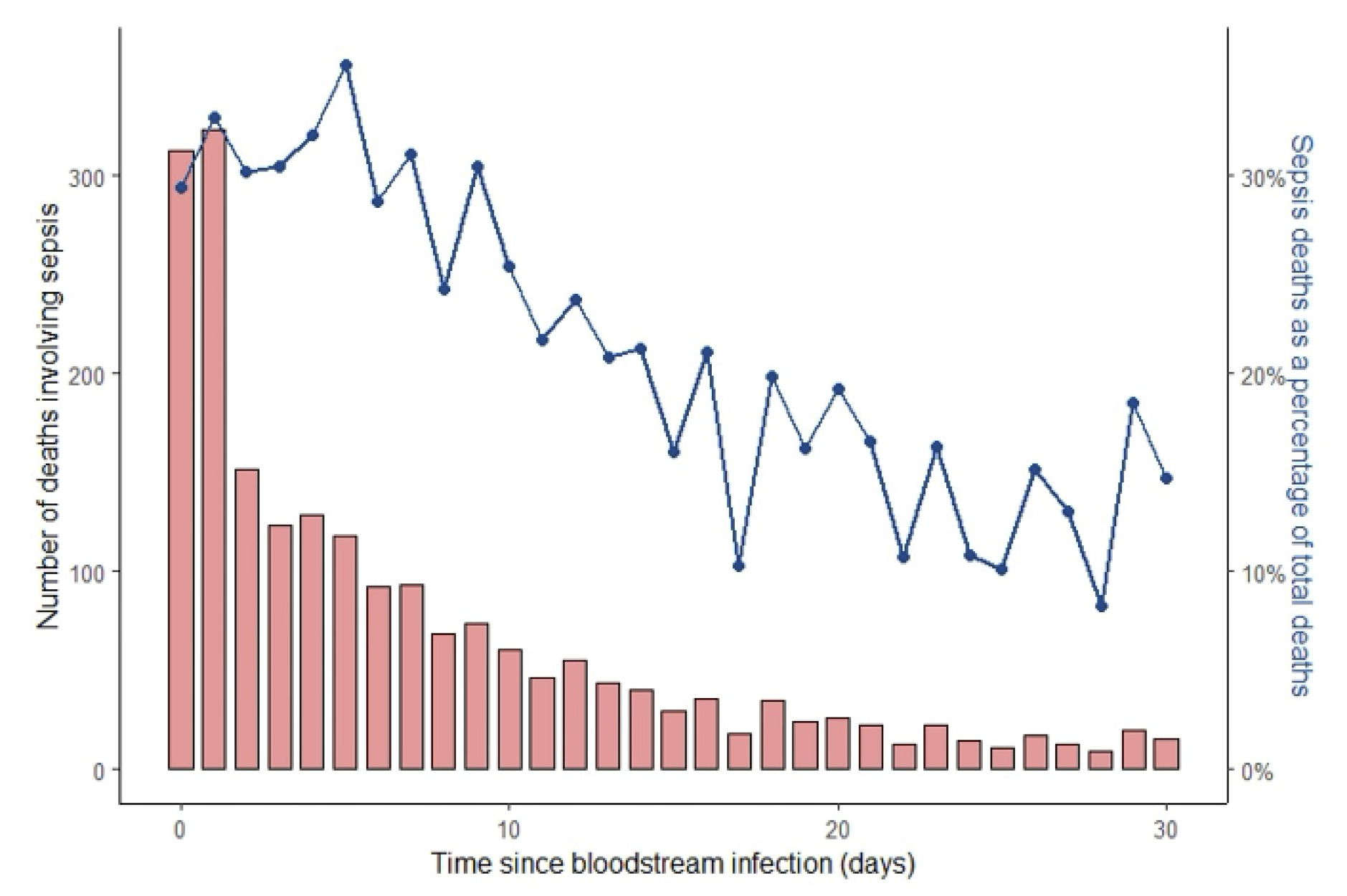

Supplement: ofae126_Supplementary_Data [file ofae126_supplementary_data.docx]
